# Initial development and psychometric testing of an instrument to measure the quality of children’s end-of-life care

**DOI:** 10.1186/1472-684X-14-1

**Published:** 2015-01-13

**Authors:** Kimberley Widger, Ann E Tourangeau, Rose Steele, David L Streiner

**Affiliations:** Lawrence S. Bloomberg Faculty of Nursing, University of Toronto, 130-155 College Street, Toronto, ON M5T 1P8 Canada; York University, 4700 Keele Street, Toronto, ON M3J 1P3 Canada; Department of Psychiatry & Behavioural Neurosciences, McMaster University, 100 West 5th, Hamilton, ON L8N 3K7 Canada; Department of Psychiatry, University of Toronto, 250 College Street, Toronto, ON M5T 1R8 Canada

**Keywords:** End-of-life, Instrument development, Mothers, Pediatrics, Quality care

## Abstract

**Background:**

The field of pediatric palliative care is hindered by the lack of a well-defined, reliable, and valid method for measuring the quality of end-of-life care.

**Methods:**

The study purpose was to develop and test an instrument to measure mothers’ perspectives on the quality of care received before, at the time of, and following a child’s death. In Phase 1, key components of quality end-of-life care for children were synthesized through a comprehensive review of research literature. These key components were validated in Phase 2 and then extended through focus groups with bereaved parents. In Phase 3, items were developed to assess structures, processes, and outcomes of quality end-of-life care then tested for content and face validity with health professionals. Cognitive testing was conducted through interviews with bereaved parents. In Phase 4, bereaved mothers were recruited through 10 children’s hospitals/hospices in Canada to complete the instrument, and psychometric testing was conducted.

**Results:**

Following review of 67 manuscripts and 3 focus groups with 10 parents, 141 items were initially developed. The overall content validity index for these items was 0.84 as rated by 7 health professionals. Based on feedback from health professionals and cognitive testing with 6 parents, a 144-item instrument was finalized for further testing. In Phase 4, 128 mothers completed the instrument, 31 of whom completed it twice. Test-retest reliability, internal consistency, and construct validity were demonstrated for six subscales: Connect With Families, Involve Parents, Share Information With Parents, Share Information Among Health Professionals, Support Parents, and Provide Care at Death. Additional items with content validity were grouped in four domains: Support the Child, Support Siblings, Provide Bereavement Follow-up, and Structures of Care. Forty-eight items were deleted through psychometric testing, leaving a 95-item instrument.

**Conclusions:**

There is good initial evidence for the reliability and validity of this new quality of end-of-life care instrument as a mechanism for evaluative feedback to health professionals, health systems, and policy makers to improve children’s end-of-life care.

**Electronic supplementary material:**

The online version of this article (doi:10.1186/1472-684X-14-1) contains supplementary material, which is available to authorized users.

## Background

Efforts to improve the quality of pediatric palliative and end-of-life care are occurring worldwide [1–4]. These efforts are hindered by the lack of a well-defined, reliable, and valid method for measuring the quality of end-of-life care. Comprehensive quality measurement requires multiple perspectives, including those of children and families [2–4]. Unfortunately, children with life-threatening conditions are sometimes unable to provide their perspective, as they may be non-verbal due to young age or the type of illness they have, or may be too weak as they near death. In this case, parents are likely to be the best proxy for the experience of their child because of the time spent together during the illness - research comparing assessments of care quality between adult patients and their family members found the greatest congruence between those who lived together or saw each other every day [5]. In addition, family-centered care is the norm in pediatrics and is based on the recognition that there are, in effect, two patients – the child and the family [6]. Therefore, parents can act as a proxy to evaluate the care provided to the child and evaluate the care that they received themselves. Ideally, these evaluations would be prospective; however, additional issues may arise with this approach, including concerns about parents being taken away from precious time with their children [7]. While there are inherent issues with quantifying experiences, proxy reports, and providing retrospective accounts of care, the bereaved parent voice is an important one to add to assessments of the quality of end-of-life care for children.

Existing research about parents’ perspectives on care provided at end-of-life indicates improvements are needed; however, the focus has been on particular disease groups (e.g., cancer) [8, 9], or locations of care (e.g., intensive care units) [10], using qualitative methods [11, 12] or instruments subjected to minimal psychometric testing [8, 9, 13]. Accurate assessment of the quality of children’s end-of-life care in a variety of situations requires identification of indicators of high quality care and development of a reliable and valid method of measurement. The purpose of this study was to develop and test an instrument to measure the quality of end-of-life care received by families who have experienced the death of a child, from the mothers’ perspective.

## Methods

The development process followed steps detailed by Streiner and Norman [14] with the instrument items designed to measure a structure, a process, or an outcome according to Donabedian’s model of quality health care [15]. Research Ethics Board (REB) approval was obtained from the University of Toronto (#23107) and at each recruitment site.

### Phase 1: literature search

Potential indicators of high quality end-of-life care were identified through a search of Medline and Cumulative Index to Nursing & Allied Health Literature (CINAHL) databases in March 2008. Search terms from 1) type of care (e.g., palliative care, bereavement care, or end-of-life care), 2) care assessments (e.g., quality of health care, needs assessment, outcome assessment, program evaluation), and 3) population (e.g., parents, mothers, fathers, family) were mapped to MeSH headings and combined. Results were limited to papers published in the preceding 10 years (1997 or later), in English, and related to children (0 – 18 years). Research papers that detailed parents’ perceptions of palliative, end-of-life, or bereavement care provided to the family by health professionals were included. Studies were not specifically assessed for scientific rigor because it was important to include the full depth and breadth of possible indicators at this early stage of instrument development [14].

### Phase 2: focus groups

Focus groups were planned, conducted, and analyzed according to procedures outlined in “The Focus Group Kit” [16]. Focus group discussions were audio recorded and transcribed verbatim. Bereaved parents received a $40 token of appreciation for participation. Parents were recruited through advertisements in newsletters and web sites associated with local bereavement organizations and support groups, as well as by word of mouth. Parents indicated interest in the study by telephone or email and were included if 1) they were the biological, step, or adoptive parent of the child who died, 2) their child who died was aged 19 years or less, and 3) the death had occurred at least one year previously. Parents who did not speak English or whose children were stillborn were excluded. Initially, only parents of children who died in hospital settings were sought. However, the child of about half the parents who responded to the advertisements had died at home. As these parents had extensive experience with hospital care prior to the death, the inclusion criteria were expanded to include them.

Parents were initially asked a broad question about what they thought was important to the provision of high quality end-of-life care for children and their families. Parents were then asked to confirm, challenge, or add to the list of indicators identified in the Phase 1 literature search. Content analysis of the transcripts was completed following each group [17]. Domains and indicators of quality pediatric end-of-life care initially identified through the literature review were revised and refined based on the focus groups.

### Phase 3: item development and refinement

Items used in previous research [8, 9] were revised and new items developed to assess the domains and associated indicators identified in Phases 1 and 2. Items reflected structures, processes, and outcomes of care [15]; however, most assessed processes and were worded to ask about care provided to the child and family or specifically to the parent. Most items had five response options on an adjectival scale ranging from “never” to “always” to reflect the frequency of occurrence of a particular aspect of care. Outcomes were assessed through ratings of satisfaction with care, overall quality, and whether the child had a ‘peaceful’ death and a ‘good’ death.

Health professionals with clinical or research expertise in pediatric end-of-life care assessed the content validity of the instrument. These judges were initially contacted through email, then sent a package with the consent form, instructions on how to assess the instrument, and the instrument. Judges rated each item on a four-point scale: one = not representative of quality care, two = needs major revisions to be representative of quality care, three = needs minor revisions to be representative of quality care, and four = representative of quality care [18]. A Content Validity Index (CVI) was computed for each item by calculating the proportion of judges who rated the item as a three or four [19]. Items receiving a rating less than 0.80 were revised. The CVI for the entire instrument was calculated by averaging the CVIs for all items [19]. An acceptable index is greater than or equal to 0.80 [18]. Judges also suggested wording changes to improve clarity, helped develop subscales by indicating which domain the item was measuring, and assessed if the instrument as a whole was a comprehensive measure of quality end-of-life care for children.

Parents who took part in the Phase 2 focus groups and gave permission to be contacted as well as parents who responded to Phase 2 advertisements after completion of the focus groups were invited to participate in Phase 3. In individual cognitive testing over a two-hour time period, parents read and re-phrased each item and/or talked through how s/he chose the item response [20]. Parents’ comments and possible changes to the instrument were incorporated from one interview into the next to see which words or questions appealed to the most parents. To assess face and content validity, parents were asked if items related to quality end-of-life care and if all aspects were included [14]. Items were revised, deleted, or added as directed by comments from health professionals and parents; parents’ comments were given greater weighting when there were discrepancies.

### Phase 4: psychometric testing

With assistance from a local investigator at each site, Phase 4 participants were recruited through 10 children’s hospitals and hospices across Canada. Although both mothers and fathers participated in focus groups and initial instrument testing, only mothers were recruited for Phase 4 as we recognized there may be differences between mothers’ and fathers’ needs and how well these needs are met. Mothers were invited to take part in the study if 1) they were the biological, step, or adoptive parent of the deceased child, 2) the child died between 6 months and 36 months prior to survey mailing, 3) the child died in hospital/hospice after minimum 24-hour admission, and 4) the child was aged 19 years or younger at time of death. Mothers were excluded if, 1) the infant died within 48 hours of birth, 2) the child died in a hospice and received only terminal care in the hospice (for example if the child was transferred from hospital to the hospice primarily for withdrawal of ventilator support), 3) they requested to have no further contact from the hospital/hospice (such as for bereavement follow-up support) 4) either parent had been implicated in the death of the child, or 5) they could not read English.

Recruitment procedures varied somewhat across sites due to local REB requirements. At all sites, contact information for eligible mothers was obtained through review of records. A healthcare professional known to the family mailed a letter with a brief description of the study to the mother. At seven sites, mothers returned an enclosed card if they did not wish to take part in the study (opt-out). If the card was not received at the originating site within three weeks, the survey package was mailed. At the other three sites, mothers returned a card indicating they wished to participate in the study (opt-in) before the survey package was mailed.

The survey package included information needed to make an informed decision about participation, the instrument itself, and an addressed, stamped return envelope. Return of the completed survey signified consent. A reminder letter was mailed through one site resulting in return of one additional survey. Reminders were not sent to non-responders at any other site due to logistical issues or REB concerns. Mothers provided contact information if they were willing to repeat the instrument in two weeks to facilitate assessment of test-retest reliability.

The instrument was designed and tested as five unique scales comprising a larger index, rather than as one single measure [21, 22]. Psychometric testing included exploratory factor analysis (EFA) to assess validity, plus internal consistency and test-retest to assess reliability. A correlation matrix of items in each subscale was generated. Items with correlations less than 0.30 or greater than 0.90 were considered for deletion [23]. The Measure of Sampling Adequacy (MSA) was calculated for each item using an anti-image correlation matrix and items less than 0.70 were considered for deletion [23]. The Kaiser-Meyer-Olkin MSA was calculated as a summary for each subscale. When the summary value (recalculated when items were deleted) was greater than 0.70, analysis continued with an assessment of factor structure [23]. EFA was conducted on each subscale using principal axis factoring and eigenvalues greater than one to generate factors. Only one factor for each subscale was expected, but when more than one factor was identified a varimax rotation was used to improve interpretation [23, 24]. Items with factor loadings less than 0.30 were considered for deletion [23]. The theoretical foundation of the instrument developed in Phase 1 and 2, the study team’s clinical and research experience with this population, and parents’ comments in Phase 3 about each item were considered in final decisions on items to retain or delete. Cronbach’s alpha was calculated for remaining items in each domain with a score between 0.70 and 0.90 indicating good internal consistency [25].

A subsample of participants completed the instrument twice. An intra-class correlation coefficient (ICC) was calculated for each revised subscale to compare scores using a two-way random effects model with a single measure and absolute agreement of scores [14]. A value greater than or equal to 0.75 was evidence of test-retest reliability [26].

## Results

### Phase 1

Sixty-seven papers reporting results of 51 separate studies were identified as relevant. The views of more than 2300 parents were represented across these studies. Five common themes/domains of high quality care specific to parent perspectives on care provided by health professionals were identified: Connect with Families, Involve Parents, Alleviate Suffering, Share Information, and Provide Bereavement Care.

### Phase 2

Three focus groups were held with 10 parents (8 biological mothers and 2 biological fathers) from 10 families, between April and June 2009. Their mean age was 44.5 years (range 38–51); 6 were married, 2 widowed, and 2 separated; and 8 had a college diploma or university degree. Nearly two thirds reported a yearly family income of greater than $60,000, 90% were Caucasian, and 40% were Catholic.

The mean age of the deceased children was 5 years (range 5 days to 15 years); 7 were girls and 3 were boys; 4 had cancer, 5 a congenital illness, and 1 a neuromuscular condition. On average, parents participated 5 years after their child had died, but the range was 1–18 years. All but two parents knew that their child was likely to die at least one month before it happened; one parent knew for less than one day. The location of the children’s deaths was equally split between home and intensive care units. Most families (70%) received some care through a specialized pediatric palliative care team.

Domains of quality of children’s end-of-life care initially derived from the literature review were confirmed. Conducting focus groups validated interpretation and synthesis of existing research. Parents highlighted areas that were in the literature but had not been identified as a specific indicator. Some indicators based on the literature review were subsumed into another indicator. For example, ‘sense of humor’, was incorporated into ‘good communication skills’ based on parents’ feedback that humor may not be appropriate for all families or at all times, but it can be a part of good communication. Additional file 1 details the associated indicators refined through focus groups.

### Phase 3

At least one item was developed for each indicator constructed through Phases 1 and 2, resulting in a 141-item instrument (plus demographic items). Of ten health professionals invited to undertake content validity testing, nine agreed and seven returned the completed instrument. Participants included two physicians, four advanced practice nurses, and one social worker. CVI scores for individual items ranged from 0.67 to 1.0; the overall CVI was 0.84.

The instrument was then reviewed by six parents (five mothers and one father); two were new to the study. Demographics were similar to those in Phase 1. Parents added items about structures of care and indicated that, while not specific to end-of-life care, the lack of availability of appropriate accommodation, food and parking significantly added to families’ distress. Other than these additions, parents felt the instrument was complete and that items related to quality end-of-life care for children. Based on health professional and parent feedback, a 144-item instrument was used for Phase 4 testing.

### Phase 4

Recruitment occurred between July and December 2010. Across sites using an opt-out process, 584 information letters were sent to eligible mothers; 68 could not be contacted; 46 opt-out cards were returned; 108 instruments were completed of 470 mailed. At sites using an opt-in process, 187 information letters were sent; 15 could not be contacted; 26 requested a copy of the instrument; and 20 were completed. In total, 128 instruments were completed (response rate 18.6%); and a further 31 were returned for reliability testing. The mean time between completions was 42.5 days (range 21–107, SD 19.4). See Tables 1 and 2 respectively for demographic information about participants and their children.

**Table 1:** **Demographic information for mothers who participated in Phase 4 (n = 128 )**

		Number (%)
Mothers’ mean age (SD)		36.5 years (8.3)
Marital status	Married	103 (80.5)
	Divorced/Separated	12 (9.4)
	Never married	11 (8.6)
	Widowed	2 (1.6)
Education	High school diploma or less	22 (17.2)
	Some college/university	24 (18.7)
	College diploma/university degree	65 (50.8)
	Post graduate degree	17 (13.3)
Income	under $25000	12 (9.7)
	$25,000 - $49,999	21 (16.1)
	$50,000 - $99,999	51 (39.5)
	$100,000 or more	44 (34.7)
Religious practice	Catholic	40 (31.4)
	Protestant	32 (25.2)
	Spiritual not Religious	26 (19.7)
	None or Atheist	19 (15.0)
	Other	11 (8.7)
Children in family (including deceased)	one child	34 (26.6)
	two children	51 (39.8)
	three or more children	43 (33.6)

**Table 2:** **Demographic information for deceased children in Phase 4 (n = 128)**

		Number (%)
Child’s mean age (SD)		4.1 years (6.2)
Girls		66 (51.6)
Boys		62 (48.4)
Primary diagnosis	Congenital malformations/chromosomal abnormalities	30 (23.4)
	Neoplasms	21 (16.4)
	Conditions originating in perinatal period	18 (14.1)
	Diseases of the nervous system	13 (10.2)
	Endocrine and metabolic diseases	9 (7.0)
	Diseases of the circulatory system	9 (7.0)
	Diseases of the digestive system	8 (6.3)
	Infectious diseases	7 (5.5)
	External causes	4 (3.1)
	Other	3 (2.3)
	Missing	6 (4.7)
Location of death	intensive care	84 (64.8)
	hospital unit	37 (28.9)
	hospice	8 (6.3)
Was a palliative care team involved?	yes	54 (42.2)
	no	51 (39.8)
	not sure	5 (18.0)

**Figure 1:**
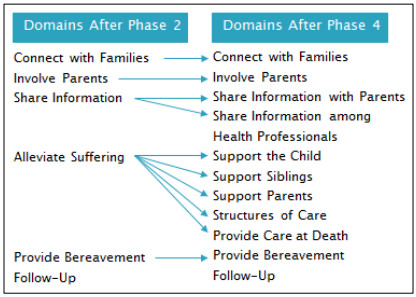
**Changes in domains from Phase 2 to 4.**

Substantial revisions were made through Phase 4 testing: 10 domains formed the revised conceptual framework and the revised instrument had 95 items on structures, processes, and outcomes (see Additional file 2). Some domains remained similar to the original conceptualization following Phase 2, while others were split in to multiple domains (see Figure 1).

The original Alleviate Suffering domain contained the largest number of items. Some items were not applicable to all respondents and some had dichotomous response options. These latter issues, coupled with the relatively low number of respondents, made EFA impossible. The items related to alleviating suffering of the child, the siblings, and the parents, as well as items about the structures of care (e.g., food, parking, accommodation), were separated into four new domains for further examination. Items in the Support the Child domain focused on evaluating the degree of suffering from a variety of symptoms. No correlations were expected between the likelihood of experiencing any given symptom and experiencing any other symptom, so a combined subscale score for symptoms did not make clinical sense and would likely have resulted in low internal consistency for a subscale. Therefore, these items remained as stand-alone items rather than being combined into an overall subscale score.

Only 80 of the 128 mothers who completed the survey had children other than the one who died. Some siblings were very young or lived far from the hospital where the child received care, so items related to siblings were sometimes not applicable. The large amount of missing data in this section resulted in a Kaiser-Meyer-Olkin MSA of 0.58, which was too low to proceed with EFA. However, the importance of support for siblings was highlighted in mothers’ comments and in findings from other researchers [27–32]. These items were retained in the instrument as stand-alone items making up the Support Siblings domain; they demonstrated good content and face validity.

Similarly, there were few correlations expected among items in the Structures of Care or Provide Bereavement Follow-up domains. The presence of any given structure or type of follow-up care did not result in a greater likelihood of any other structure or follow-up being present. However, findings from the initial study phases, as well as recent findings [29, 33–38] from others, indicated a need for greater attention to these structures of care and to bereavement follow-up when assessing quality of end-of-life care for children. Items comprising the Structures of Care and Provide Bereavement Follow-up domains were retained as stand-alone items with good content and face validity.

The remaining items from the original Alleviate Suffering domain were further subdivided into two subscales (Support Parents and Provide Care at Death) based on EFA results. Overall, the analysis resulted in six subscales (Connect With Families, Involve Parents, Share Information With Parents, Share Information Among Health Professionals, Support Parents, and Provide Care at Death,) where item responses are combined to produce a summary score for the subscale. There was good evidence for the test-retest reliability of these 6 subscales with ICCs ranging from 0.81 to 0.90. See Table 3 for further psychometric information. Four other domains were not associated with a subscale score and consist of stand-alone items only: Support the Child, Support Siblings, Structures of Care, and Provide Bereavement Follow-up. Content and face validity of all stand-alone items was demonstrated in study Phase 3.

**Table 3:** **Exploratory factor analysis (EFA) and reliability testing results by subscale**

Subscale	Number of items	Kaiser-Meyer-Olkin MSA ^a^	EFA item loadings	Cronbach’s alpha	ICC ^b^
Connect with families	16	0.94	0.53-0.85	0.96	0.90
Involve parents	8	0.80	0.50-0.89	0.88	0.82
Share information with parents	9	0.91	0.50-0.79	0.90	0.81
Share information among health professionals	4	0.91	0.59-0.80	0.86	0.88
Support parents	11	0.85	0.51-0.86	0.92	0.83
Provide care at death	7	0.78	0.24-0.84	0.76	0.81

^a^MSA = Measure of Sampling Adequacy.

^b^ICC = Intra-class Correlation Coefficient.

## Discussion

Overall, there was initial evidence for the reliability and validity of six subscales and content validity of four additional domains when tested with bereaved mothers. The instrument is applicable to end-of-life care in a broad range of illnesses, ages, and hospital/hospice settings. While most instruments used in research with similar pediatric populations have been developed with parental input and assessed for face and content validity [8, 9, 13], to our knowledge our instrument is the first to be subjected to this degree of psychometric testing.

As the content of the instrument items was initially developed based on existing research with parents, the 10 domains that arose from EFA are also consistent with existing literature. In a more recent metasummary of the needs of patients and families in pediatric palliative care, 10 domains were identified: interactions with staff, health care delivery and accessibility, information needs, bereavement needs, psychosocial needs, spiritual needs, pain and symptom management, cultural needs, siblings’ needs, and decision making [39]. While domain names differed, there was a great deal of content overlap with the exception of structures of care, which is missing from the metasummary, and the specific focus on decision-making, which is not separately included in our instrument but is somewhat incorporated into the Involve Parents domain. The metasummary findings support retention of items related to pain and symptom management, siblings, and bereavement care; however, additional work is required to re-work these items to develop subscales in the future.

EFA was the only method to assess validity beyond content and face validity in this initial psychometric testing of the instrument. There are no comparable instruments for use in pediatrics, but in adult palliative care similar instruments to measure quality of care from the perspective of bereaved family members have assessed construct validity through hypothesis testing. For example, construct validity of the Quality of Dying and Death (QODD) instrument was supported by higher scores when deaths occurred at home and symptom scores were lower [40]. Similarly, construct validity of the Caregiver Evaluation of the Quality of End-Of-Life Care (CEQUEL) Scale was supported by higher scores with home hospice enrollment, longer length of inpatient hospice stays, and lower levels of bereaved caregiver regret and psychological trauma [41]. The state of the science in pediatric palliative care made it difficult to identify similar hypotheses about the direction of scores with enough evidence that would support the validity of the instrument if the hypotheses were supported. Additional assessment of validity will be important in future testing. Our assessment supported the test-retest reliability of our instrument, which has not previously been assessed in other pediatric or adult based instruments, and indicates the stability of mothers’ responses over time.

The overall response rate was low and varied at each participating site (range 6.8-34.8%), as well as by method of recruitment (11.6% opt-in versus 21.3% opt-out). Response rates in other studies with bereaved parents have ranged from 17-80% [8, 13]. When testing a new instrument, a wide range of responses and diverse experiences is more important than a high response rate [14]. Although there was little diversity in participating mothers’ demographics, there was a wide range of ages and diagnoses of the deceased children and deaths occurred in 10 different hospitals/hospices. Additionally, participants used the full range of possible item response options, indicating the needed variation in the quality of care provided was sufficiently present for psychometric testing. Response rates may be improved in future testing by offering multiple, flexible ways for this population to take part in research, including telephone or in-person interviews, a web-based survey, or a mailed out survey [42]. Since the number of items was reduced from 144 to 95 based on psychometric testing, parents may find the shorter version less burdensome to complete, resulting in higher response rates.

The instrument was tested only with bereaved mothers. Recruiting fathers in this type of research has been difficult in the past [43], but that should not preclude attempts to do so. As fathers were involved in Phases 1–3, the revised instrument will be tested with them as an important next step in the development process.

## Conclusions

Although there are areas of the instrument that need further revision and testing, this research is a significant step forward in providing a valid and reliable way to include the voice of the bereaved parent in assessing quality of end-of-life care for children. The major contribution of the study is identification of 10 domains important to high quality care, as well as a method for measurement. Attention to measurement and improvement of these structures and processes of care will assist health professionals, health systems, and decision-makers to support children and families in the midst of their suffering and promote the best possible outcomes.

## Electronic supplementary material

Additional file 1: **Phase 2 Domains and Indicators of Quality End-of-Life Care.** (PDF 156 KB)

Additional file 2: **Revised Quality of Children’s End-of-Life Care Instrument.** (PDF 219 KB)

## Abbreviations

REB: Research ethics board
EFA: Exploratory factor analysis
MSA: Measure of sampling adequacy
ICC: Intra-class correlation coefficient
PPC: Pediatric palliative care
SD: Standard deviation
CVI: Content validity index.
