# Supplementary material for: Initial development and psychometric testing of an instrument to measure the quality of children’s end-of-life care
Source: BMC Palliat Care. 2015 Jan 13;14:1. doi: 10.1186/1472-684X-14-1 (PMC4305389; doi:10.1186/1472-684X-14-1)
Supplement: Supplementary file 1 — Additional file 1: Phase 2 Domains and Indicators of Quality End-of-Life Care. (PDF 156 KB) [file 12904_2014_240_MOESM1_ESM.pdf]

Phase 2 Domains and Indicators of Quality End-of-Life Care

Domain Name	Indicator Type	Indicator
Connect With Families	Process	Accessible and consistent health professionals involved in care<br>Connection between health professionals and all family members<br>Health professionals have good communication skills<br>Health professionals demonstrate sensitivity and compassion<br>Health professionals are 'human'<br>'Fit' between health professional and family<br>Individualized care<br>Parent/child did not feel avoided or abandoned<br>Trust between health professionals and family<br>Small acts of human kindness demonstrated
	Outcome	Satisfaction with connections between health professionals and family
Involve Parents	Structure	Space for parent to be physically present with child throughout care
	Process	Health professionals seek out and listen to parent's views/concerns<br>Health professionals respect parent's wishes<br>Parent feels supported and strengthened in their role as parent<br>Parent's desired involvement in child's physical care is respected<br>Parent given opportunity to be present for life-threatening events
	Outcome	Satisfaction with level of involvement in child's care
Alleviate Suffering	Structure	Quiet and private space offered for all family members
	Process	Offer options and opportunity to access services, fulfill wishes<br>Family's choice of pace is respected<br>Child's physical, emotional, social and spiritual symptoms are assessed and treated<br>Parents'/siblings' emotional, social, practical and spiritual needs are anticipated, assessed, and treated<br>Hope is supported (but not at all costs)<br>Options for timing/location of death provided if life support withdrawn<br>Sensitive/respectful care of the body
	Outcome	Child's death free from suffering and respects wishes of child/family<br>Satisfaction with treatment of all child's symptoms<br>Satisfaction with support of parents and siblings
Share Information	Process	Health professionals give complete, truthful, consistent, and concrete information<br>Information included "the big picture"<br>Health professionals assess and respect amount of information desired by parents<br>Possibility of death raised and discussed by health professionals<br>Information given about what to expect at the time of death<br>Information needs anticipated by health professionals<br>Adequate time provided to share information<br>Information offered appropriately to child by health professionals<br>Information is appropriately shared among health professionals<br>Team/family meetings held
	Outcome	Satisfaction with information given to parents and child<br>Satisfaction with information shared among health professionals

<table>
<tr><th>Domain Name</th><th>Indicator Type</th><th>Indicator</th></tr>
<tr><td rowspan="3">Provide Bereavement Care</td><td>Structure</td><td>Memorial service held by hospital</td></tr>
<tr><td>Process</td><td>Grief and community resource information provided
Anticipate and offer to collect mementos
Assistance with funeral arrangements offered
Autopsy discussed sensitively
Organ/tissue donation discussed sensitively
Autopsy results received in a timely manner
Follow-up meeting with health professionals offered
Parents did not feel abandoned after the death
Health professionals offer continued contact with the family</td></tr>
<tr><td>Outcome</td><td>Satisfaction with follow-up support provided through the hospital</td></tr>
</table>
