# Supplementary material for: Initial development and psychometric testing of an instrument to measure the quality of children’s end-of-life care
Source: BMC Palliat Care. 2015 Jan 13;14:1. doi: 10.1186/1472-684X-14-1 (PMC4305389; doi:10.1186/1472-684X-14-1)
Supplement: Supplementary file 2 — Additional file 2: Revised Quality of Children’s End-of-Life Care Instrument. (PDF 219 KB) [file 12904_2014_240_MOESM2_ESM.pdf]

Revised Quality of Children's End-of-Life Care Instrument

Connect With Families Domain

Connect With Families Subscale Items

1. How often did health professionals communicate well with you and your family? *Never / Rarely / Sometimes / Mostly / Always*
2. How often were health professionals sensitive to you and your family's feelings? *Never / Rarely / Sometimes / Mostly / Always*
3. How often did you feel a close connection to the health professionals who cared for your child? *Never / Rarely / Sometimes / Mostly / Always*
4. How often was there a "good fit" between health professionals and your family? *Never / Rarely / Sometimes / Mostly / Always*
5. How often did you feel health professionals accepted you and your family without judging you? *Never / Rarely / Sometimes / Mostly / Always*
6. How much did you trust the health professionals caring for your child? *Not at all / Not very much / Somewhat / Mostly / Completely*
7. How often did health professionals act as if they were better than you? *Never / Rarely / Sometimes / Mostly / Always*
8. How often did health professionals show you their "human" side? *Never / Rarely / Sometimes / Mostly / Always*
9. How often did health professionals treat your child as a unique person? *Never / Rarely / Sometimes / Mostly / Always*
10. How often did health professionals treat you as a unique person? *Never / Rarely / Sometimes / Mostly / Always*
11. How often did you experience "acts of kindness" from health professionals while you were in hospital? *Never / Rarely / Sometimes / Mostly / Always*
12. How often was it easy to contact the health professionals caring for your child? *Never / Rarely / Sometimes / Mostly / Always*
13. How often were health professionals open to talking about your concerns? *Never / Rarely / Sometimes / Mostly / Always*
14. How often was there at least one team member working consistently with your family? *Never / Rarely / Sometimes / Mostly / Always*
15. How often did you know which health professional was in charge of your child's care? *Never / Rarely / Sometimes / Mostly / Always*
16. Did you and your family ever feel avoided or abandoned by health professionals before your child's death? *Never / Rarely / Sometimes / Mostly / Always*

Connect With Families Outcome Item

Overall, how satisfied were you with your relationships with the health professionals who cared for your child before his/her death? *Completely dissatisfied / Mostly dissatisfied / Somewhat dissatisfied / Neither satisfied nor dissatisfied / Somewhat dissatisfied / Mostly satisfied / Completely satisfied*

Involve Parents Domain

Involve Parents Subscale Items

1. How often did health professionals ask for your opinions or concerns about your child? *Never / Rarely / Sometimes / Mostly / Always*
2. How often did you feel trusted as the "expert" on your child? *Never / Rarely / Sometimes / Mostly / Always*
3. How often did health professionals respect your wishes for your child's care? *Never / Rarely / Sometimes / Mostly / Always*
4. How often did health professionals help you to feel that you were doing the best you could for your child? *Never / Rarely / Sometimes / Mostly / Always*
5. How often did health professionals support you in your role as a parent? *Never / Rarely / Sometimes / Mostly / Always*
6. How often were you as involved in your child's care as you wanted to be? *Never / Rarely / Sometimes / Mostly / Always*
7. How often were you given a choice whether or not to be with your child during difficult procedures or life threatening events (for example if cardiopulmonary resuscitation [CPR] was done, right after surgery, during tests etc.)? *No difficult procedures or life threatening events / Never / Rarely / Sometimes / Mostly / Always*
8. How often were health professionals available to support you during difficult procedures or life threatening events? *Never / Rarely / Sometimes / Mostly / Always*

Involve Parents Outcome Item

Overall, how satisfied were you with your involvement in your child's care during the last week or days of his/her life? *Completely dissatisfied / Mostly dissatisfied / Somewhat dissatisfied / Neither satisfied nor dissatisfied / Somewhat dissatisfied / Mostly satisfied / Completely satisfied*

Share Information With Parents Domain

Share Information With Parents Subscale Items

1. How often did health professionals let you choose the amount of information that you wanted shared with you? *Never / Rarely / Sometimes / Mostly / Always*
2. How often did health professionals give you the right amount of information about your child's condition? *Never / Rarely / Sometimes / Mostly / Always*
3. How often did health professionals seem to know what information you might need before you even asked? *Never / Rarely / Sometimes / Mostly / Always*
4. How often did health professionals give information to you and your family that was confusing? *Never / Rarely / Sometimes / Mostly / Always*
5. How often did you feel health professionals gave truthful information to you and your family about your child? *Never / Rarely / Sometimes / Mostly / Always*
6. How often did health professionals show you your child's test results, X-rays, or scans when you wanted to see them? *I did not want this kind of information / Never / Rarely / Sometimes / Mostly / Always*

7. How often did health professionals talk about "the big picture" for your child's condition (for example not just how his/her heart or lungs were working)? *Never / Rarely / Sometimes / Mostly / Always*
8. How often did health professionals provide enough time when talking with you and your family so you did not feel rushed? *Never / Rarely / Sometimes / Mostly / Always*
9. How often did you take part in family and team meetings about your child's care? *Never / Rarely / Sometimes / Mostly / Always*

Share Information With Parents Outcome Item

Overall, how satisfied were you with the information shared between you and the health professionals caring for your child? *Completely dissatisfied / Mostly dissatisfied / Somewhat dissatisfied / Neither Satisfied nor Dissatisfied / Somewhat dissatisfied / Mostly satisfied / Completely satisfied*

Share Information Among Health Professionals Domain

Share Information Among Health Professionals Subscale Items

1. How often was the information you received about your child the same from one health professional to the next? *Never / Rarely / Sometimes /* Mostly / Always
2. From your perspective, how often was information appropriately shared among health professionals? *Never / Rarely / Sometimes / Mostly / Always*
3. How often were you the one to tell health professionals the medical details of your child's condition because they didn't seem to know? *Never / Rarely / Sometimes / Mostly / Always*
4. How often did it seem health professionals planned together so they were all working towards the same goals for your child's care? *Never / Rarely / Sometimes / Mostly / Always*

Share Information Among Health Professionals Outcome Item

Overall, how satisfied were you with the information shared among all the health professionals caring for your child? *Completely dissatisfied / Mostly dissatisfied / Somewhat dissatisfied / Neither satisfied nor dissatisfied / Somewhat dissatisfied / Mostly satisfied / Completely satisfied*

Support the Child Domain

Support the Child Stand Alone Items

1. How often did health professionals look at all the needs of your child (physical, emotional, social, developmental, and spiritual needs)? *Never / Rarely / Sometimes / Mostly / Always*
2. How often did health professionals appropriately involve your child in talks about his/her illness? *Never / Rarely / Sometimes / Mostly / Always / Not applicable (for example if child was too young or unable to communicate)*

During your child's last week or days of life while in the hospital, how much would you say your child seemed to suffer from each of the following symptoms?

3. Pain *Not at all / A little / Somewhat / A lot / Constantly / Unsure*
4. Nausea or vomiting *Not at all / A little / Somewhat / A lot / Constantly / Unsure*
5. Breathing Difficulties *Not at all / A little / Somewhat / A lot / Constantly / Unsure*
6. Bleeding *Not at all / A little / Somewhat / A lot / Constantly / Unsure*

7. Seizures *Not at all / A little / Somewhat / A lot / Constantly / Unsure*
8. Sadness *Not at all / A little / Somewhat / A lot / Constantly / Unsure*
9. Anxiety / Worry *Not at all / A little / Somewhat / A lot / Constantly / Unsure*
10. Fear *Not at all / A little / Somewhat / A lot / Constantly / Unsure*

Support the Child Outcome Items

1. Overall, how satisfied were you with the information shared between health professionals and your child? *Child too young / Completely dissatisfied / Mostly dissatisfied / Somewhat dissatisfied / Neither satisfied nor dissatisfied / Somewhat dissatisfied / Mostly satisfied / Completely satisfied*
2. Overall, which of the following describes the amount of suffering your child had from his/her symptoms? Would you say he / she suffered: *Not at all / A little / Somewhat / A lot / Constantly / Unsure*
3. Overall, how satisfied were you with support given to ease your child's suffering by health professionals at the hospital? *Completely dissatisfied / Mostly dissatisfied / Somewhat dissatisfied / Neither satisfied nor dissatisfied / Somewhat satisfied / Mostly satisfied / Completely satisfied*

<u>Support Siblings Domain</u>

Support Siblings Stand-Alone Items

1. How often did health professionals provide the right amount of overall support to your other children during the time your child was in hospital? *No other children / Never / Rarely / Sometimes / Mostly / Always*
2. How often did health professionals guide you on how you could support your other children while your child was in hospital? *No other children / Never / Rarely / Sometimes / Mostly / Always*
3. How often did health professionals allow and encourage your other children to visit when they wished? *No other children / Never / Rarely / Sometimes / Mostly / Always*

Support Siblings Outcome Item

Overall, how satisfied were you with support given to your other children by health professionals at the hospital? *No other children / Completely dissatisfied / Mostly dissatisfied / Somewhat dissatisfied / Neither satisfied nor dissatisfied / Somewhat dissatisfied / Mostly satisfied / Completely satisfied*

<u>Support Parents Domain</u>

Support Parents Subscale Items

1. How often did health professionals provide the right amount of support for your practical needs (for example access to food, a place to stay, transportation)? *Never / Rarely / Sometimes / Mostly / Always*
2. How often did health professionals provide the right amount of support for your emotional needs (for example feeling listened to, accepting your feelings)? *Never / Rarely / Sometimes / Mostly / Always*

3. How often did health professionals provide the right amount of support for your spiritual needs? *Never / Rarely / Sometimes / Mostly / Always*
4. How often did health professionals provide the right amount of support for your social needs (for example feeling cared about, maintaining relationships with your family and friends)? *Never / Rarely / Sometimes / Mostly / Always*
5. Some parents describe having a health professional who was able to "coach" or guide them during their child's end of life care. This health professional was someone who was able to anticipate family needs, prepare and support the family throughout the process, and give helpful options and suggestions about things to do. How often was there a health professional involved with your family who acted as this type of guide? *Never / Rarely / Sometimes / Mostly / Always*
6. How often did health professionals try to fulfill any of your family's special requests or wishes? *No special requests made / Never / Rarely / Sometimes / Mostly / Always*
7. How often did health professionals assist you to get any additional services that you might need? *Never / Rarely / Sometimes / Mostly / Always*
8. How often was your family given private time with your child before he / she died? *Never / Rarely / Sometimes / Mostly / Always*
9. How often did health professionals allow and encourage extended family members / friends to visit when you wished? *Never / Rarely / Sometimes / Mostly / Always*
10. How often did health professionals support your hopes for your child? *Never / Rarely / Sometimes / Mostly / Always*
11. How often did health professionals find the right balance in helping you hope for the best possible outcome (cure or longer life) for your child while also making plans in case that outcome did not happen? *Never / Rarely / Sometimes / Mostly / Always*

Support Parents Outcome Item

Overall, how satisfied were you with support given to you by health professionals at the hospital? *Completely dissatisfied / Mostly dissatisfied / Somewhat dissatisfied / Neither satisfied nor dissatisfied / Somewhat dissatisfied / Mostly satisfied / Completely satisfied*

Provide Care at Death Domain

Provide Care at Death Subscale Items

1. Once it was clear that your child was likely to die, were you given choices about where this might happen (for example going home, going to a hospice, or moving to a more private room or area of the hospital)? *Yes / No / Can't Remember / No choices possible as my child died very suddenly*
2. Did health professionals respect cultural / religious / spiritual practices that you and your family wanted around the time of your child's death? *No respect at all / A bit respectful / Somewhat respectful / Very respectful /Extremely respectful*
3. Did health professionals respect your wishes for which family members or friends you wanted with you when your child died? *No respect at all / A bit respectful / Somewhat respectful / Very respectful /Extremely respectful*

4. Did health professionals respect your wishes for which health professionals you wanted with you when your child died? *No respect at all / A bit respectful / Somewhat respectful / Very respectful /Extremely respectful*
5. How helpful was it to have a health professional with you when your child died? *No health professional was with us / Not at all helpful / A little helpful / Somewhat helpful / Very helpful / Extremely helpful*
6. Were health professionals sensitive and respectful when caring for your child's body after death? *Not at all / Not very sensitive / Somewhat sensitive/ Mostly sensitive / Completely sensitive*
7. Some parents want to stay with their child for a long time after he / she dies, while others want to leave the hospital fairly quickly. Were you given as much time as you wanted with your child after he / she died? *Yes / No*

Provide Care at Death Outcome Items

1. Some people use the word peaceful to describe the atmosphere at the time of a person's death. Which of the following best describes the atmosphere at the time of your child's death? *Not at all peaceful / A bit peaceful / Somewhat peaceful / Very peaceful / Extremely peaceful*
2. Although the death of a child is always a tragedy, some parents are able to describe their child's dying process as a good death. All things considered, would you say your child experienced a "good death"? *Disagree strongly / Disagree somewhat / Neither agree nor disagree / Agree somewhat / Agree strongly*

<u>Provide Bereavement Follow-up Domain</u>

Provide Bereavement Follow-up Subscale Items

1. Did health professionals suggest and offer to create mementos (for example: lock of hair, pictures, hand/foot prints) of your child? *Yes / No / Don't remember*
2. Did health professionals offer to help you make any arrangements for funerals or other religious customs? *Yes / No / Don't remember*
3. About how many contacts with health professionals from the hospital have you had since your child died? _____
4. Did someone from the hospital offer you information about your grief? *Yes / No / Don't remember*
5. Did someone from the hospital/hospice offer you information about your other children's grief?
   Don't have other children / Yes / No / Don't remember
6. Did someone from the hospital offer you information about community resources available close to your home? *Yes / No / Don't remember*
7. Were you offered a follow-up meeting with health professionals to discuss what happened at the time of your child's death and/or the autopsy results (if one was done)? *Yes / No / Don't remember*

Provide Bereavement Follow-up Outcome Item

Overall, how satisfied were you with the support provided through the hospital in the months after your child died? *Completely dissatisfied / Mostly dissatisfied / Somewhat dissatisfied / Neither satisfied nor dissatisfied / Somewhat dissatisfied / Mostly satisfied / Completely satisfied*

Structures of Care Domain

Structures of Care Stand Alone Items

1. How often was there enough room for you to be with your child while in hospital? *Never / Rarely / Sometimes / Mostly / Always*
2. How often was there a comfortable place for you to sleep at or near the hospital? *Never / Rarely / Sometimes / Mostly / Always*
3. How often were there playrooms or other child-friendly spaces for your other children while you were at the hospital? *No other children / Never / Rarely / Sometimes / Mostly / Always*
4. How often was food readily available for your family while you were at the hospital? *Never / Rarely / Sometimes / Mostly / Always*
5. Would you say the food at the hospital was affordably priced? *Not at all / A little / Somewhat / Very / Extremely*
6. How often were you easily able to find parking at the hospital? *Never / Rarely / Sometimes / Mostly / Always*
7. Would you say parking at the hospital was affordably priced? *Not at all / A little / Somewhat / Very / Extremely*

Overall Quality

Overall, how would you describe the quality of end-of-life care provided to your child and family by health professionals from the hospital? *Poor / Fair / Good / Very Good / Excellent*
